# Supplementary material for: Global unleashing of transcription elongation waves in response to genotoxic stress restricts somatic mutation rate
Source: Nat Commun. 2017 Dec 12;8:2076. doi: 10.1038/s41467-017-02145-4 (PMC5727188; doi:10.1038/s41467-017-02145-4)
Supplement: Supplementary file 1 — Supplementary Information [file 41467_2017_2145_MOESM1_ESM.pdf]

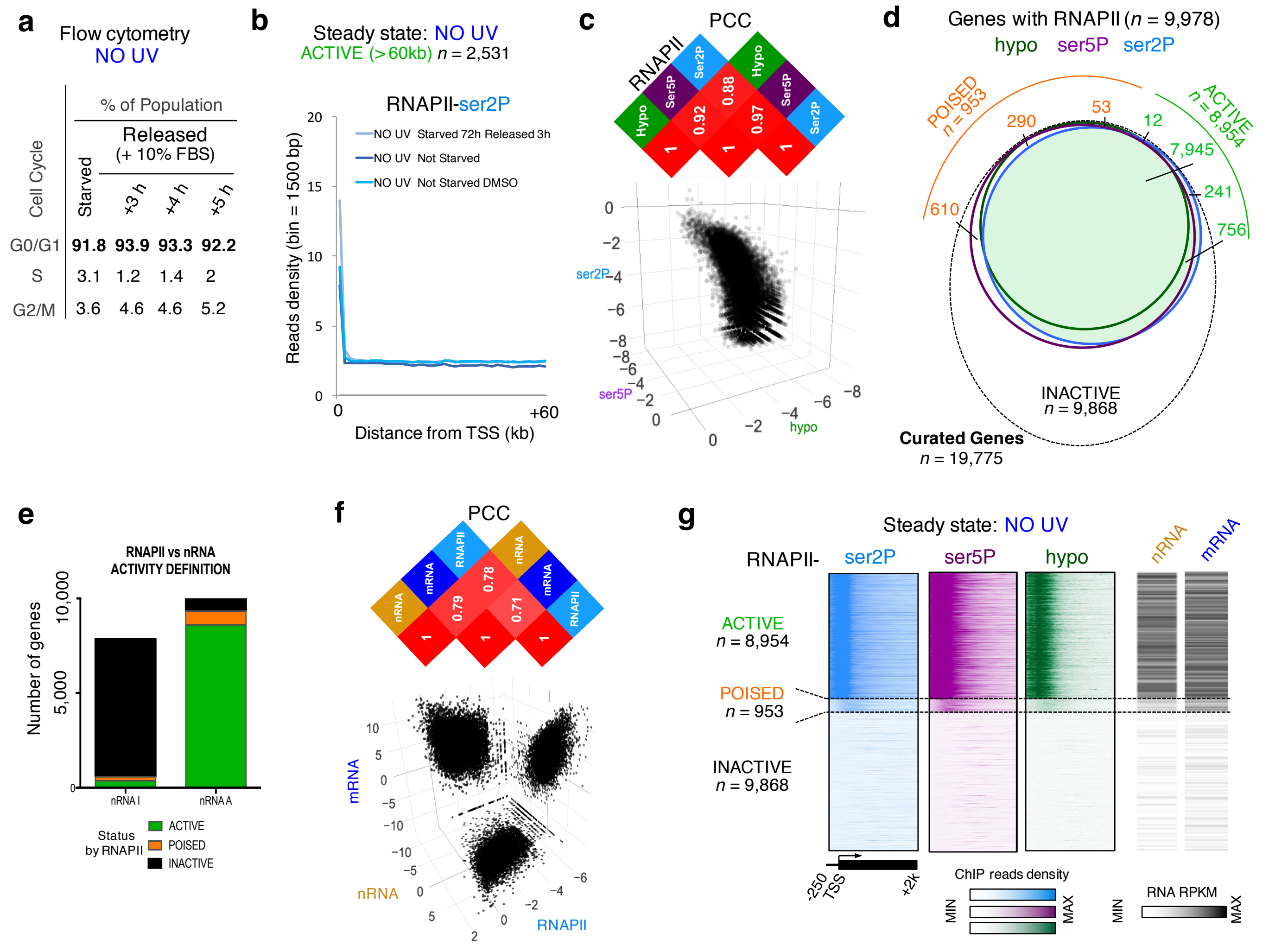

Supplementary Fig. 1 Determination of constitutive gene expression status before stress (steady-state or NO UV). **a** Flow cytometry analysis of cell cycle. Percentages of cells in each cell cycle phase are shown for cells starved (+0.5% FBS) for 72 h and for cells released in complete medium (+10% FBS) for 3 to 5 h. **b** Average plots of RNAPII-ser2P ChIP-seq read densities on active genes (see **d**) larger than 60 kb from TSS to TSS + 60 kb before irradiation (NO UV). We compare cells starved (+0.5% FBS) for 72 h and released in complete medium (+10% FBS) for 3 h, with cells not starved, and cells not starved and treated with DMSO for 16 h prior release in normal medium for 1 h. **c** Correlation analysis of RNAPII-hypo, -ser5P and -ser2P ChIP-seq Densities around promoters before irradiation (Dp = density (reads per million) on promoter proximal regions, from -250 bp to +100 bp around TSS). Pearson's Correlation Coefficients (PCC) were calculated and reported on the correlation heat map. Intense red color represents a stronger correlation score. **d** Gene expression status for the curated gene list (see Supplementary Methods) was defined by intersecting genes with MACS2 peaks (on promoter proximal regions) for RNAPII-hypo (dark green), -ser5p (purple) and -ser2P (blue) ChIP-seq. For higher stringency, RNAPII-ser2P list was further filtered by selecting only the genes with Dp > 0.7 rpm. RNAPII- ser2P containing genes were considered as active (solid green), while the union of RNAPII-ser5P and -hypo genes that did not overlap -ser2P genes were defined as poised. The rest of the genes were considered as inactive. See Supplementary Data 2 and 3. **e** Comparison of gene expression status determined by RNAPII ChIP-seq in **c** with the status determined by nascent RNA-seq (nRNA-seq status: A = active for genes with RPKM greater than an arbitrary threshold located between the two peaks of the Kernell distribution; I =Inactive for the rest of the genes, see also Methods). **f** Same as in (**c**) for RNAPII-ser2P (Dp), nRNA-seq (RPKM gene) and mRNA-seq (RPKM exon). **g** Heatmaps illustrating the distribution of RNAPII isoforms reads before UV irradiation, as aligned at individual (rows) promoter-proximal regions (-250 bp to +2 kb relative to TSS) and categorised by gene expression status, as defined in (**d**). Corresponding nascent (nRNA) and processed mature (mRNA) RNA expression levels (RPKM gene and exon, respectively) are shown for each row.

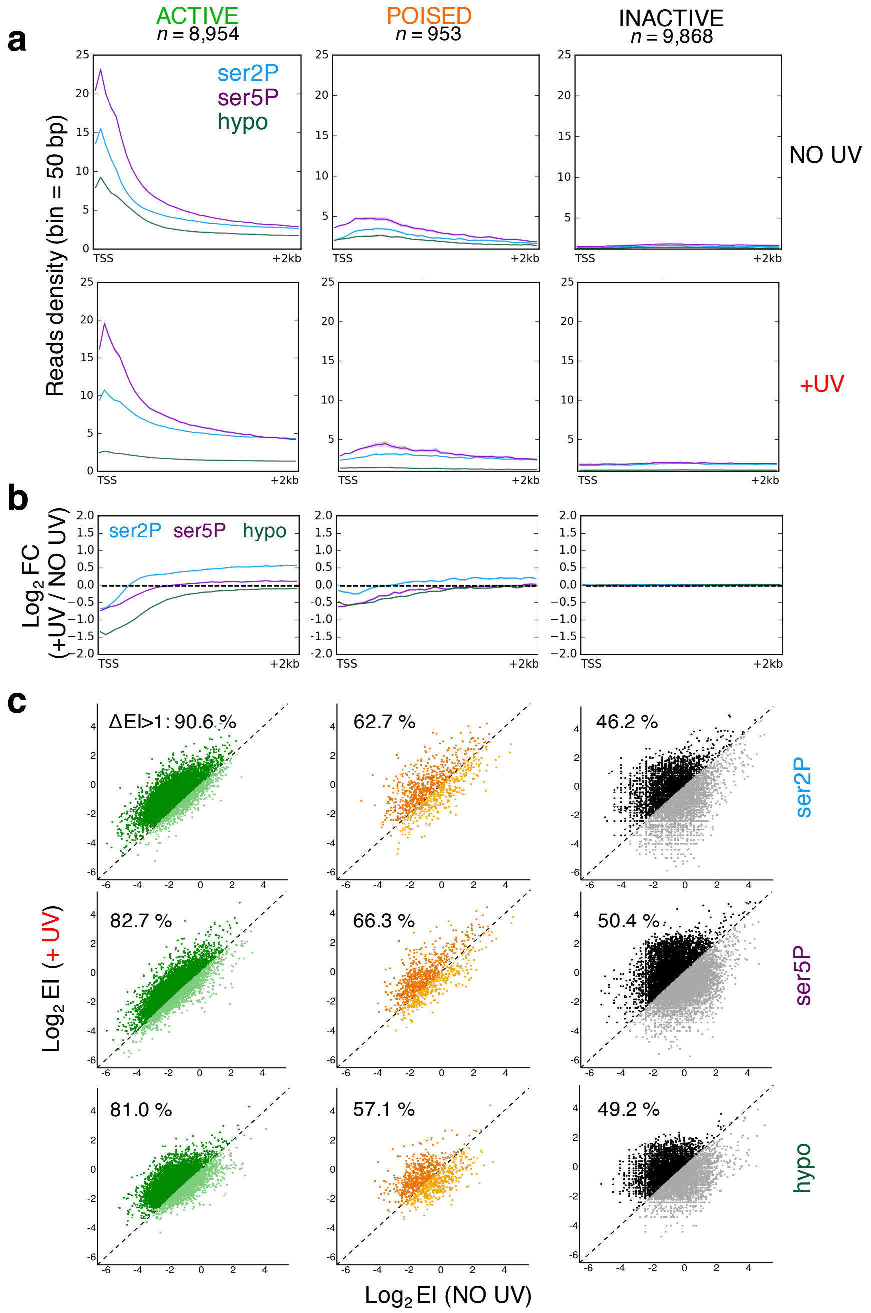

d

RNAPII-		%ΔEI>1 ACTIVE	%ΔEI>1 POISED	%ΔEI>1 INACTIVE	A vs P Chi-Square	A vs P p-val
ser2P	0.5h 8J/NO UV	89.6	61.8	45.8	584.13	<.0001
ser2P	1 h 8J/NO UV	90.6	62.7	46.2	625.21	<.0001
ser2P	2 h 8J/NO UV	89.2	61.4	48.1	569.17	<.0001
ser2P	6 h 8J/NO UV	87.0	57.0	47.8	577.97	<.0001
ser2P	48 h 8J/NO UV	70.1	50.9	52.1	146.6	<.0001
ser5P	0.5h 8J/NO UV	82.7	66.3	50.4	149.12	<.0001
ser5P	48h 8J/NO UV	40.9	48.9	48.8	22.35	<.0001
hypo	1.5h 8J/NO UV	81.0	57.1	49.2	291.46	<.0001
hypo	48h 8J/NO UV	46.0	45.9	47.3	0.01	0.9203

%ΔEI >1: 100 – 50 – 0; χ^2: MAX – MIN

Supplementary Fig. 2 Genome–wide reorganisation of RNAPII isoforms during UV stress response. **a** Average plots of read densities for RNAPII-ser2P, - ser5P and -hypo on active, poised and inactive genes from TSS to TSS + 2 kb, before (NO UV) and after (+UV) irradiation. **b** Same as in (**a**), but showing the difference in binding profiles of RNAPII isoforms after UV irradiation (8 J m^{-2}) (Log_2 FC= (read density +UV)/ (read density NO UV)). **c** Individual comparison of escape index (EI, see Fig. 1b) before (NO UV) and after UV (+UV) for all active, poised and inactive genes. Percentages of genes with increased RNAPII escape from promoter-proximal pausing (PPP) regions after UV (ΔEI >1, dark green dots above the y=x dotted line) are shown. **d** Summary heatmap representing the changes in percentages (blue=0% to red=100%) of genes with ΔEI > 1 for time series analysis of RNAPII-hypo, -ser5P, and -ser2P and for gene expression categories defined in (**c**). Chi-square test (X^2) compares the number of genes in active and poised categories for each condition and determines if the observed number of genes with ΔEI > 1 differs from expected value purely by chance. *P* values are indicated on the right and are considered significant if < 0.01. Data complied with normal distribution and tests were performed with the tool available at http://vassarstats.net/newcs.html.

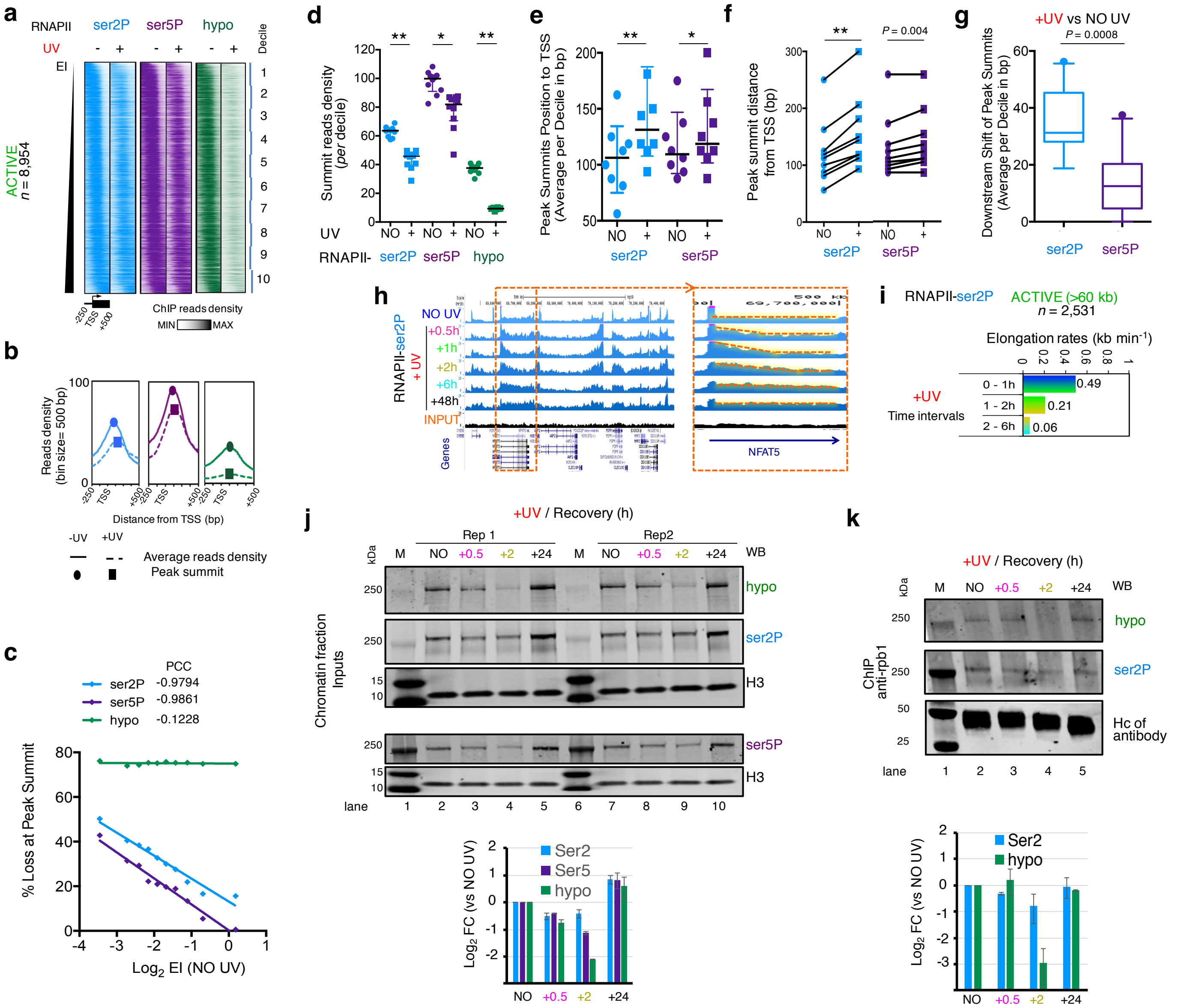

Supplementary Fig. 3 RNAPII reorganisation around PPP regions and in chromatin. **a** Heatmaps illustrating the distributions of hypo (*dark green*), ser5P (*purple*) and ser2P (*light blue*) -RNAPII reads, before (-) and after (+) UV irradiation (8 J m^{-2}), aligned at individual (rows) PPP regions (-250 bp; +500 bp of TSS) of all active genes ranked by increasing EI (as in Fig. 1e). Deciles are indicated. **b** Average plots of read densities shown in (**a**). Peak summits distance from TSS are defined at maximum reads density. **c** Correlation plot between RNAPII-ser2P EI (NO UV) and percentage of signal loss at peak summits after UV irradiation for samples and conditions defined in (**a**). PCCs are indicated. **d** Comparison of ChIP-seq summit read densities. Average values calculated for deciles defined in **a** are plotted. Median value is shown with black horizontal bar and error bars represent the interquartile range. $**P < 0.0001$, $*P < 0.0005$ (paired t-test (two-tailed)). **e** Analysis of ChIP-seq peak summits average positions relative to TSS per decile, as defined in (**a**), for RNAPII-ser2P and –ser5P. Median value is shown with black horizontal bar and error bars represent interquartile range. **: $P < 0.0001$, *P: < 0.0005 (paired t-test (two-tailed) was used for variance analysis). **f** Pairwise analysis of changes in decile's average peak summits distance to TSS (NO vs + UV irradiation for RNAPII-ser2P and -ser5P. **: $P < 0.0001$ (paired t-test (two-tailed)). **g** Analysis of shifts in ChIP-seq peak summits positions relative to TSS for conditions defined in (**a**) for RNAPII-ser2P and –ser5P. Median value is shown with horizontal bar and boxes represent the 10-90 percentile. $P = 0.0008$ (paired t-test (two-tailed)). **h** UCSC snapshot of RNAPII-ser2P binding profiles before and after UV stress. Tracks are normalised to the minimum total number of reads sequenced across samples. Input is shown in black. Genes are shown at the bottom. The orange box highlights one representative gene displaying a time-dependent progressing wave of elongating RNAPII. **i** Average ($n = 2{,}531$) elongation rates (kb/min) for RNAPII-ser2P ChIP-seq were calculated from the average wave front positions determined in Fig. 1e. Gradients of colors reflect time intervals. **j**-**k**, Input- and ChIP-western blot analysis of RNAPII-CTD phosphorylation patterns with quantification (bottom, mean (±SEM) Log2 FC as compared to NO UV) (hypo: dark green, ser5P: purple and ser2P: light blue) before (NO) and after UV irradiation (8 J m^{-2}). Input (**j**) amounts represent 5% of the starting material of cross-linked chromatin fraction (Histone H3 was used as a representative loading control). For ChIPs (**k**), equal amounts of anti-RPB1 antibody (raised against the N-terminus of RNAPII main subunit and therefore recognising all RNAPII isoforms: pan-RNAPII) were incubated with equal amounts of cross-linked chromatin. The material was resolved by 4-12% Bis-Tris Gel (NuPage), transferred and immuno-blotted with indicated antibodies, see also Methods. Hc indicates the heavy chain. Data shown reflects 2 independent experiments.

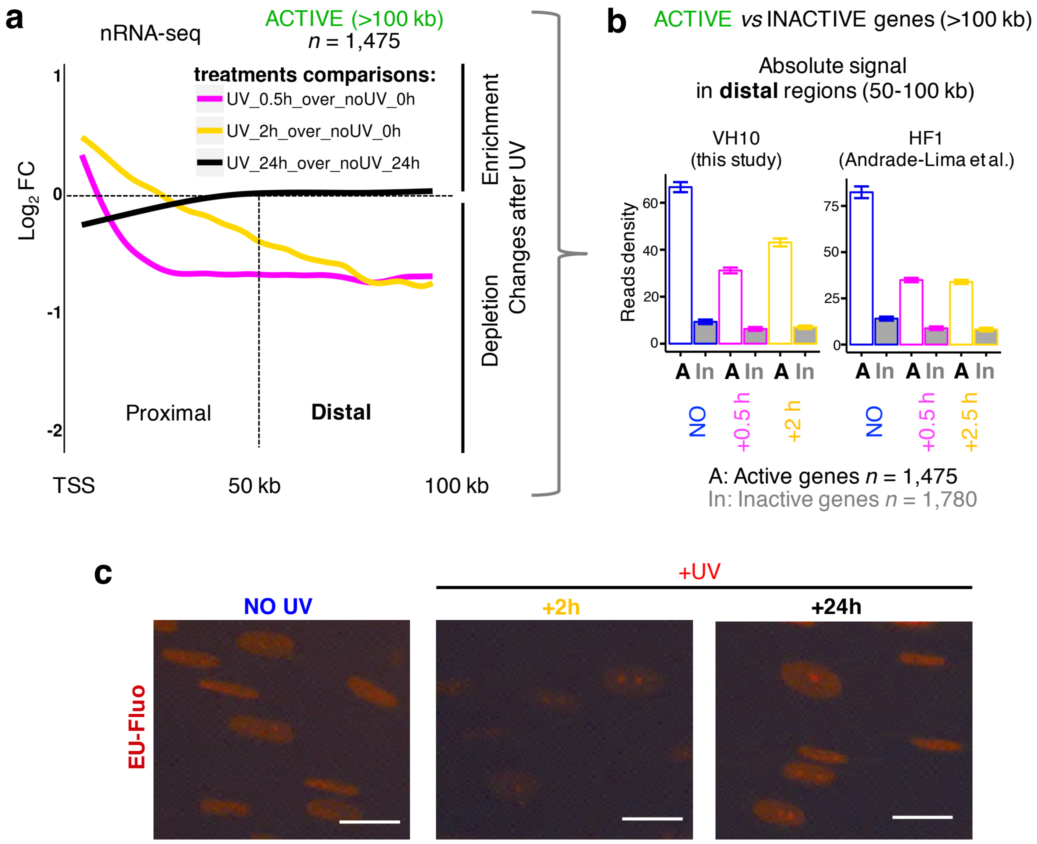

Supplementary Fig. 4 Characterisation of the UV-dependent global and reversible changes of nascent RNA synthesis. **a** Average plots showing the changes in nRNA-seq read densities after UV irradiation (15 J m^{-2}) in starved-released VH10 cells (nRNA was labeled with EU for 10 min before indicated time, see also Supplementary Methods). Gene length of genes larger than 100 kb from transcription start sites (TSS) to TSS +100 kb. During the early recovery period (0.5 to 2 h), depleted Log_2 FC scores show an increase in nRNA synthesis for 5' regions and a reduction for the rest of gene bodies. **b** Bar plots showing the mean (±SEM) of absolute density (rpm) of nRNA-seq reads in distal regions (50-100kb downstream of TSS) of active and inactive genes longer than 100 kb, as detected for VH10 (samples defined in (a)) and for HF1 cells (hTert immortalised wild type human fibroblasts) using the nascent RNA-seq data (Bru-seq) generated by Andrade-Lima *et al.*[1] Note: gene expression status was very well correlated between VH10 and HF1 cells (not shown). **c** Fluorescent nascent RNA labeling with EU (EU-Fluo) was performed on starved-released VH10 cells UV-irradiated (15 J m^{-2}), or not, according to manufacturer's guidelines using Alexa Fluor 594 Azide (Molecular Probes) for the 'click-it' reaction (see also Supplementary Methods). Data shown is representative of at least 2 independent experiments. Scale bar: 20 µm.

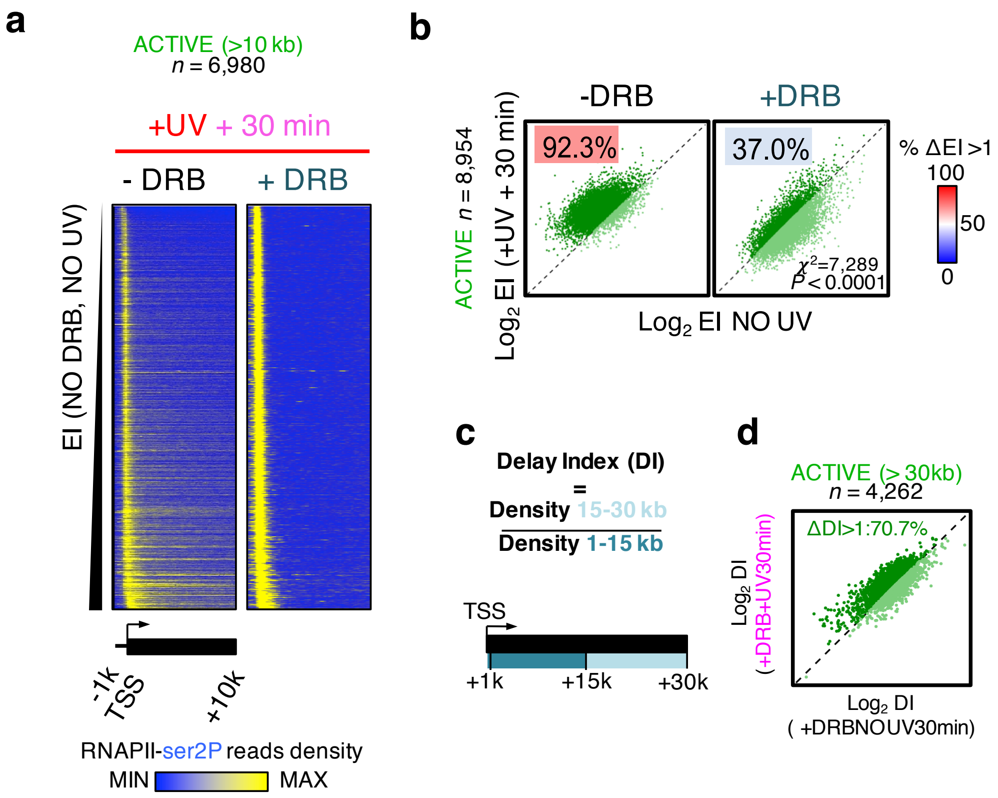

Supplementary Fig. 5 Characterisation of DRB-inhibition effect on global UV-dependent RNAPII reorganisation. **a** Heatmaps illustrating the distribution of ser2P-RNAPII reads, aligned at individual (rows) active genes larger than 10kb from TSS -1 kb to TSS +10 kb and ranked by increasing constitutive EI (as in Fig. 1d), after UV irradiation (20 J/m^2) in the presence (+) or not (-) of DRB inhibitor. **b** Individual comparison of constitutive EI (NO UV) with EI after UV (+ and – DRB) for RNAPII-ser2P and for all active genes. Percentage of genes with increased escape after UV (ΔEI > 1, dark green dots) is shown color-coded. X^2 test determines if the observed number of genes with ΔEI > 1 significantly differs between treatments. Data complied with normal distribution and tests were performed with the tool available at http://vassarstats.net/newcs.html. **c** Delay Index (DI) of pri-elongating RNAPII represent the ratio of reads density between gene regions ranging from TSS + 15 kb to TSS + 30 kb (pale turquoise) and regions ranging from TSS + 1 kb to TSS + 15kb (dark turquoise) for genes longer than 30 kb. **d** Individual comparison of DI in the presence of DRB without UV treatment or with UV treatment (ΔDI=DI(+DRB+UV)/DI(+DRB-UV)) for all active genes after 30 min of recovery. Percentage of genes delayed after UV (ΔDI>1, dark green dots) is shown.

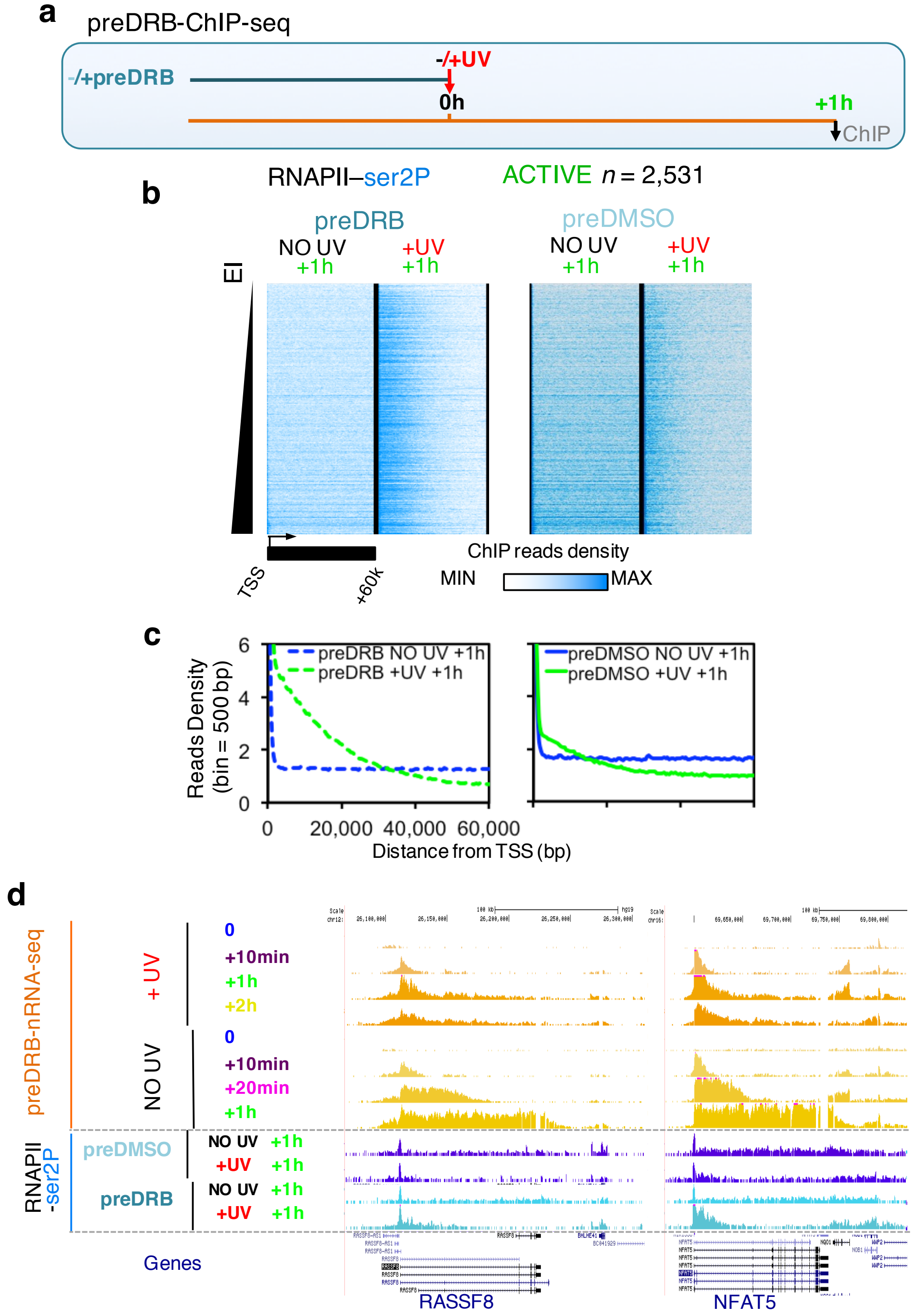

Supplementary Fig. 6 Characterisation of global UV-dependent RNAPII and nascent transcription reorganisation after DRB-release. **a** Scheme of DRB-releasing methodology for ChIP-seq (preDRB-ChIP-seq). Cells were pre-incubated with DRB (100 µM) for 12 h, washed briefly with PBS and mock (NO UV) or UV-irradiated (20 J m^{-2}) and incubated in normal conditions to follow transcription recovery. RNAPII-ser2P was purified from cross-linked chromatin isolated after 1 h of recovery from UV or mock treatment. See also Methods. **b** Heatmaps of distribution of RNAPII-ser2P preDRB-ChIP-seq reads, aligned at individual (rows) active genes (length > 60 kb) from TSS to TSS +60 kb) and ranked by increasing EI (as determined before UV without DRB in Fig. 1e), with (+) or without UV irradiation (NO UV) 1 h after release from DRB or DMSO. **c** Average plots of read densities for conditions defined in (**b**). **d** preDRB-nRNA-seq and preDRB-ChIP-seq (RNAPII-ser2P) UCSC snapshots at two representative loci for conditions defined in Fig. 2d and in (**a**) respectively. Tracks are normalised to the minimum total number of reads sequenced across samples. See Supplementary Methods for details. Genes are shown at the bottom and are orientated from left to right.

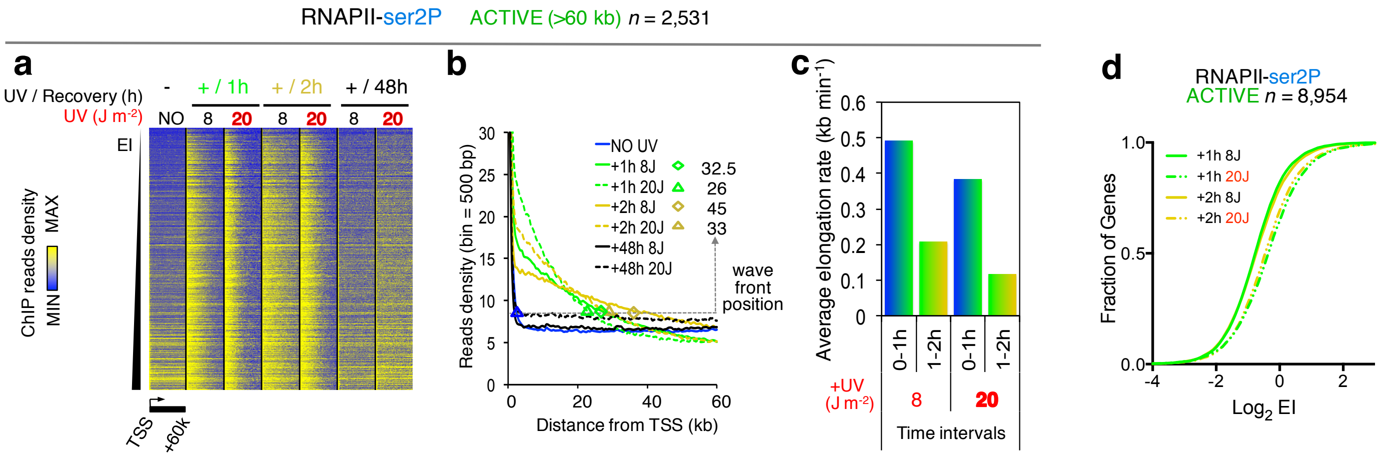

Supplementary Fig. 7 Global delays in RNAPII progression is UV-dose dependent. **a** Heatmaps illustrating the distribution of RNAPII-ser2P reads aligned at individual (rows) active genes (solid green) larger than 60 kb, from TSS to TSS +60 kb, and ranked by increasing constitutive EI (see Fig. 1d) for NO UV (-) and for different doses of UV irradiation (8 J m^{-2} vs 20 J m^{-2}) after 1 (*bright green*), 2 (*gold*) and 48 (*black*) h of recovery. **b** Average plot of read densities shown in (**a**). Wavefront positions are indicated at arbitrary threshold representing the transition state (*de novo* wave to pri-elongating RNAPII). **c** Average (n = 2,531) elongation rates (kb min^{-1}) were determined for two time intervals for conditions defined in (**a**) and were calculated from the average wave front positions determined in (**b**). **d** RNAPII-ser2P EIs for increasing UV irradiation doses after 1 and 2 h recovery plotted as ECDF of active genes.

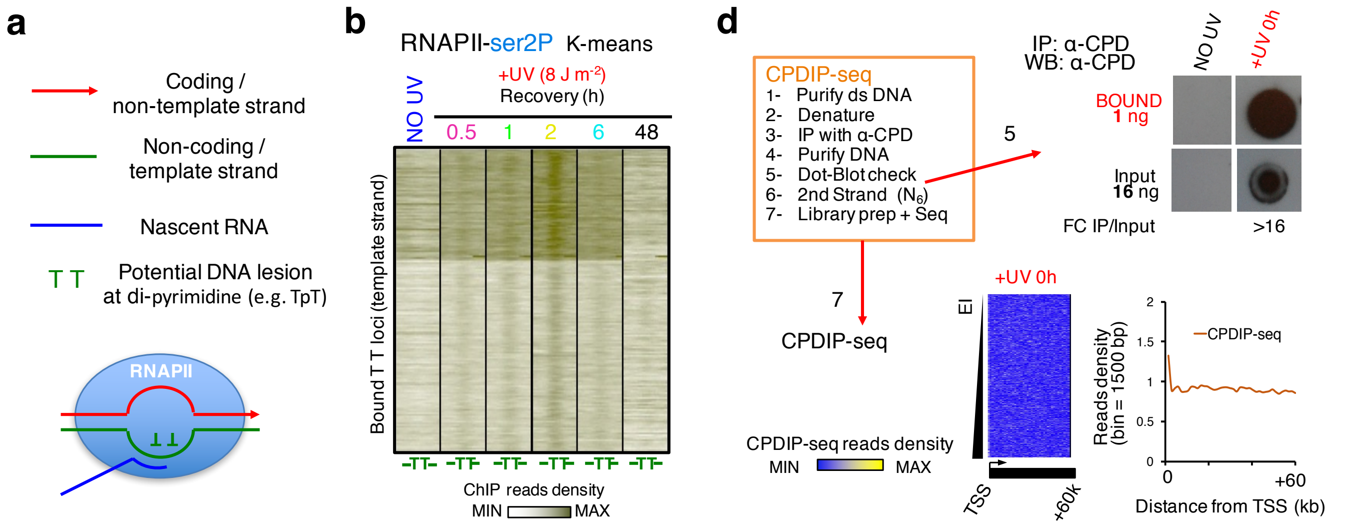

c

CLUSTER	Number of TT	Number of Exon Start	Distance to TSS (bp) Start	Distance to TSS (bp) End	Wavefront +1h	Wavefront +2h	Wavefront +6h
I	4215	1249	3000	10000	Upstream	Upstream	Upstream
II	7917	1565	10001	32500	Upstream	Upstream	Upstream
III	2243	500	32501	45000	Downsteam	Upstream	Upstream
IV	1955	481	45001	59500	Downsteam	Downsteam	Upstream
V	3256	1114	59501	100000	Downsteam	Downsteam	Downsteam
VI	5271	1627	100001	1150817	Downsteam	Downsteam	Downsteam

Supplementary Fig. 8 RNAPII accumulates transiently at damaged TT loci during the recovery process. **a** A scheme describes the definition of Non-overlapping TT loci detected on the template strand. We kept all TT loci on the template strand of our curated gene list (see Supplementary Fig. 1) if within active gene borders and if the distance to next or previous TT (on template strand), was greater than 70 bp. See Supplementary Data 3. **b** Heatmaps illustrating the distribution of RNAPII-ser2P reads aligned around intragenic TT loci, as defined in (**a**), before (NO UV) and after UV irradiation (+UV, 8J m^{-2}) and clustered by K-means, see also Methods. Two representative clusters of RNAPII-bound TT are shown. **c** Summary heatmap indicating the number of elements, border positions (bp), and relative location in comparison to wave front positions (see Fig. 1e) for clusters of TT and exon start loci analysed in Fig. 4 and Supplementary Fig. 9. **d** CPDIP-seq was developed to assess the global genomic distribution of CPD UV lesions. The box indicates the outline of the methodology (see Methods for details). *5* shows a dot-blot analysis confirming enrichment for CPD-containing DNA after IP with anti-CPD antibody. Note: the proportion (x16) in blotted amounts of INPUT and IP (BOUND) material was necessary to avoid hyper-saturation of detected signal in IP. *7* shows the distribution (left: heatmap; right: average profile) of CPDIP-seq reads, aligned at individual (rows) genes from TSS to TSS +60 kb, for DNA isolated straight after irradiation.

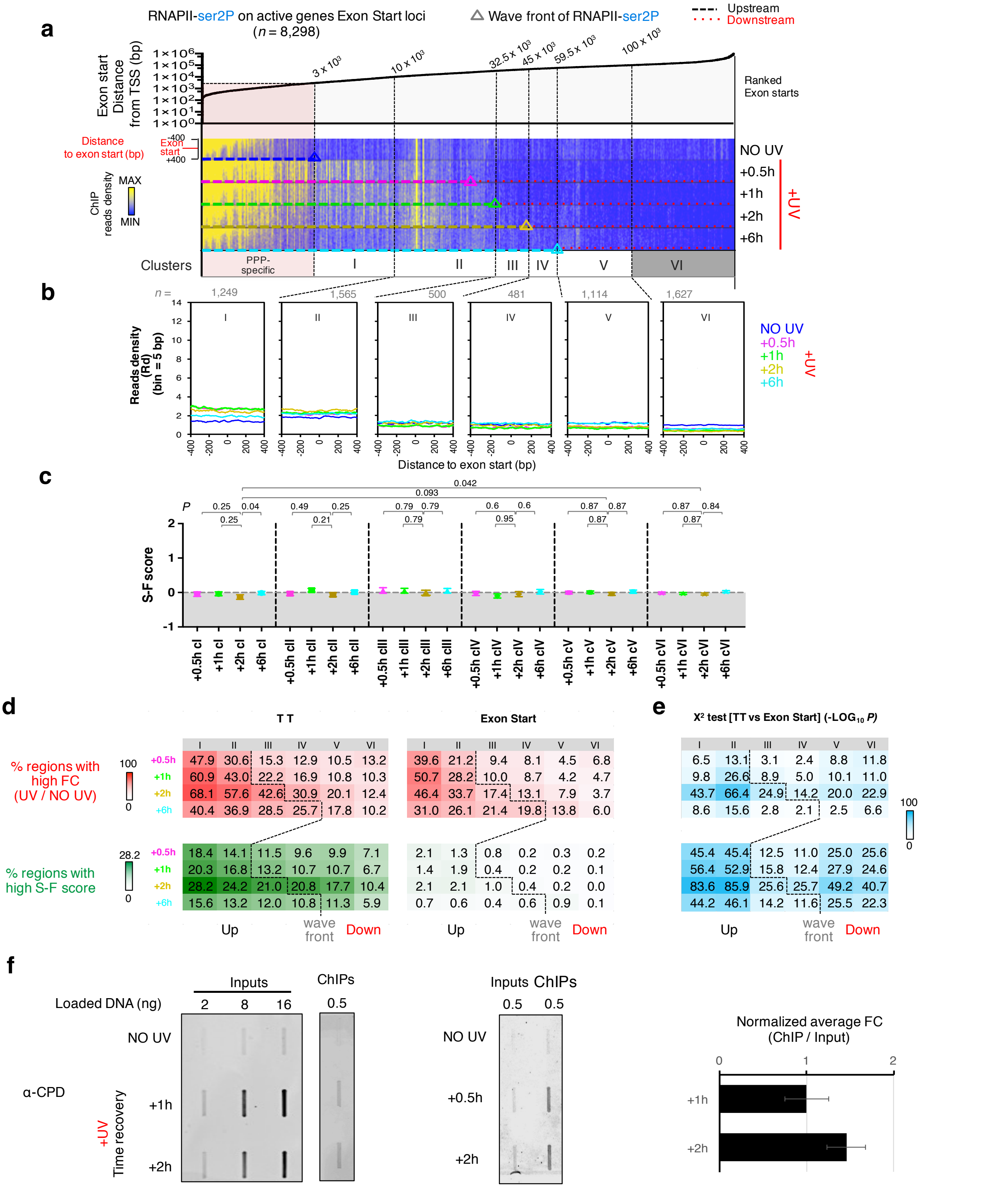

Supplementary Fig. 9 *De novo* wave release upon UV genotoxic stress does not promote specific stalling of RNAPII at exon start loci. **a** Heatmaps illustrating the distribution of RNAPII-ser2P reads aligned around a representative subset of exon start loci spanning active genes (see also Methods and Supplementary Fig. 8c) before (NO UV) and after UV irradiation (+UV, 8J m^{-2}) and sorted from left to right by increasing distances relative to TSS. Exon start loci were then clustered according to their position relative to RNAPII-ser2P wave front positions. Upstream clusters (*i.e.* I, II, III for + 2h) are enriched for *de novo* released RNAPII, and Downstream clusters (i.e. Clusters IV, V, VI for + 2h) for pri-elongating molecules (see Fig. 4 and Supplementary Fig. 8c). Loci near PPP-specific RNAPII signal were not considered for analysis. **b** Average plots of read densities (Rd) mapped in (**a**) for individual clusters (*n* is indicated). **c** Average S-F score (± SEM) representing the difference between Rd calculated around exon start positions and Rd at flanking genomic positions. Pairwise comparisons are shown, and *P* values were calculated by the two-sided Wilcoxon rank-sum test. *P* were considered Non-Significant (N.S.) if > 0.05. **d** *Top:* Heatmap representing the proportion of the regions (*per* cluster) with high Fold Changes (FC > 2) in Rd in the whole region analysed (-400;+400 bp from center). *Bottom:* Heatmap representing the proportion of regions (*per* cluster) with high S-F scores (> threshold = average[S-F]$_{exon\ start}$ + 3*SD[S-F]$_{exon\ start}$), as calculated for TT loci (see Fig. 4. The *Dotted line* indicates the location of the wave front. Clusters of loci located Upstream (Up) or Downstream (Down) are indicated (*left and right from the dotted line*, see (**a**), Supplementary Fig. 8c and Methods). **e** Heatmaps showing the differences in Chi-

square (X^2) test calculated for FC and S-F score. We compare the number of regions detected for TT and for Exon Start loci in each condition and in matching cluster. *P*-values reflect if observed values differ from expected value purely by chance. **f** Dot-blot analysis of RNAPII-ser2P ChIP-enriched and Input DNA before and after UV irradiation (8 J m^{-2}). DNA was denatured, and equal amounts of ChIP and Input DNA were hybridised to a nitrocellulose membrane before incubation with anti-CPD antibody (Cosmobio, dilution at 1:500). CPD-enrichment was quantified in at least two replicates. Fold Changes (FC) between ChIP and Input (corrected for NO UV signal) are represented in the bar graph (mean (±SEM)).

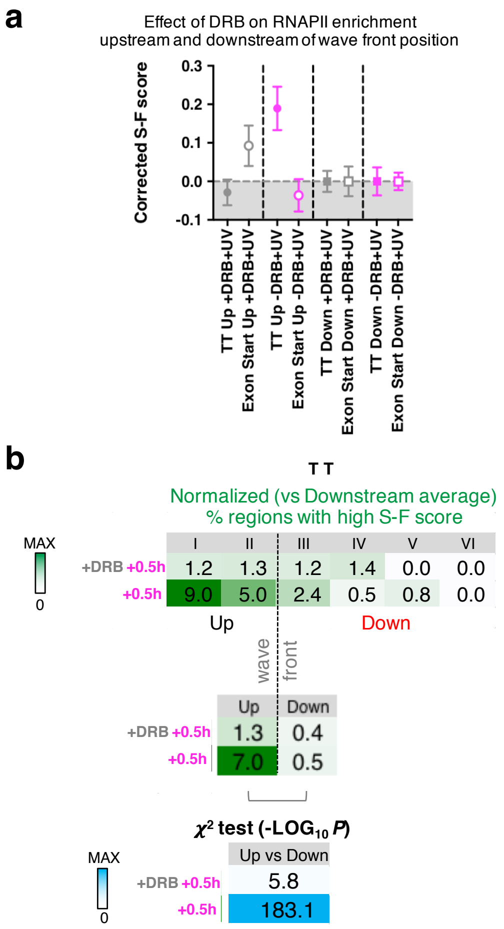

Supplementary Fig. 10 Inhibition of global *de novo* wave release by DRB cancels the boost in RNAPII stalling at TT loci located upstream of the theoretical wave front position (observed without drug). **a** Average (± SEM) S-F scores were calculated in the DRB-ChIP-seq (see Fig. 2) data and compared to scores obtained in normal conditions (see Fig. 4) for loci located Upstream (Up) or Downstream (Down) of the wave front calculated in the respective condition. All scores were corrected by inferring the average S-F score calculated for all Down loci. **b** Detailed and summary analysis of the effect of DRB on differences in proportion of regions displaying high S-F scores. Chi-square test (X^2) compares the number of genes in Up and Down categories for each condition and determines if observed number of regions with high S-F scores differs from the expected value purely by chance.

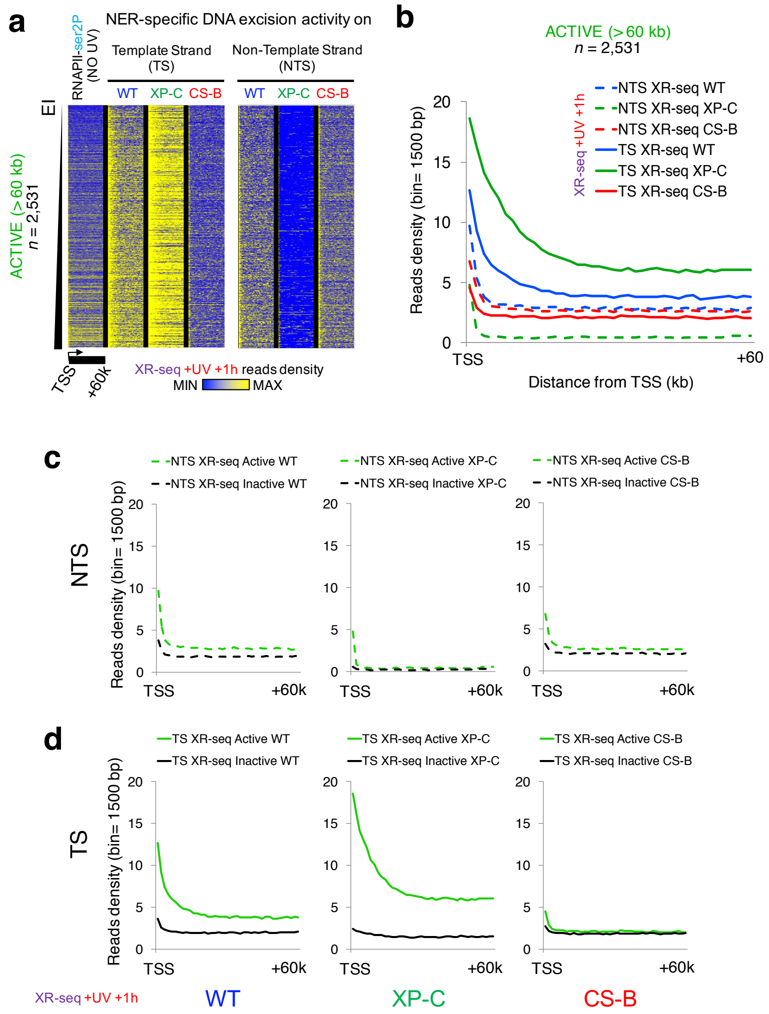

Supplementary Fig. 11 Global *de novo* wave release correlates with increased DNA excision activity in active genes on both strands. **a** Heatmaps illustrating the distribution of excised DNA reads (XR-seq[1]) 1 h after irradiation on each strand separately (Template Strand = TS and Non-Template Strand = NTS, see also Supplementary Fig. 8a for definition), as aligned at individual (rows) active genes larger than 60 kb from TSS to TSS +60 kb, and ranked by increasing constitutive RNAPII-ser2P EI (see Fig. 1d), in TC-NER and GG-NER competent cells (WT), in GG-NER deficient cells (XP-C) and in TC-NER deficient cells (CS-B). Heatmaps of RNAPII-ser2P ChIP-seq in WT cells before UV are shown on the left. **b** Average profile plots of read densities mapped in (**a**). **c**, **d** Comparison of average profile plots of XR-seq read densities between active and inactive genes for TS (**c**) and NTS (**d**). See Methods for details.

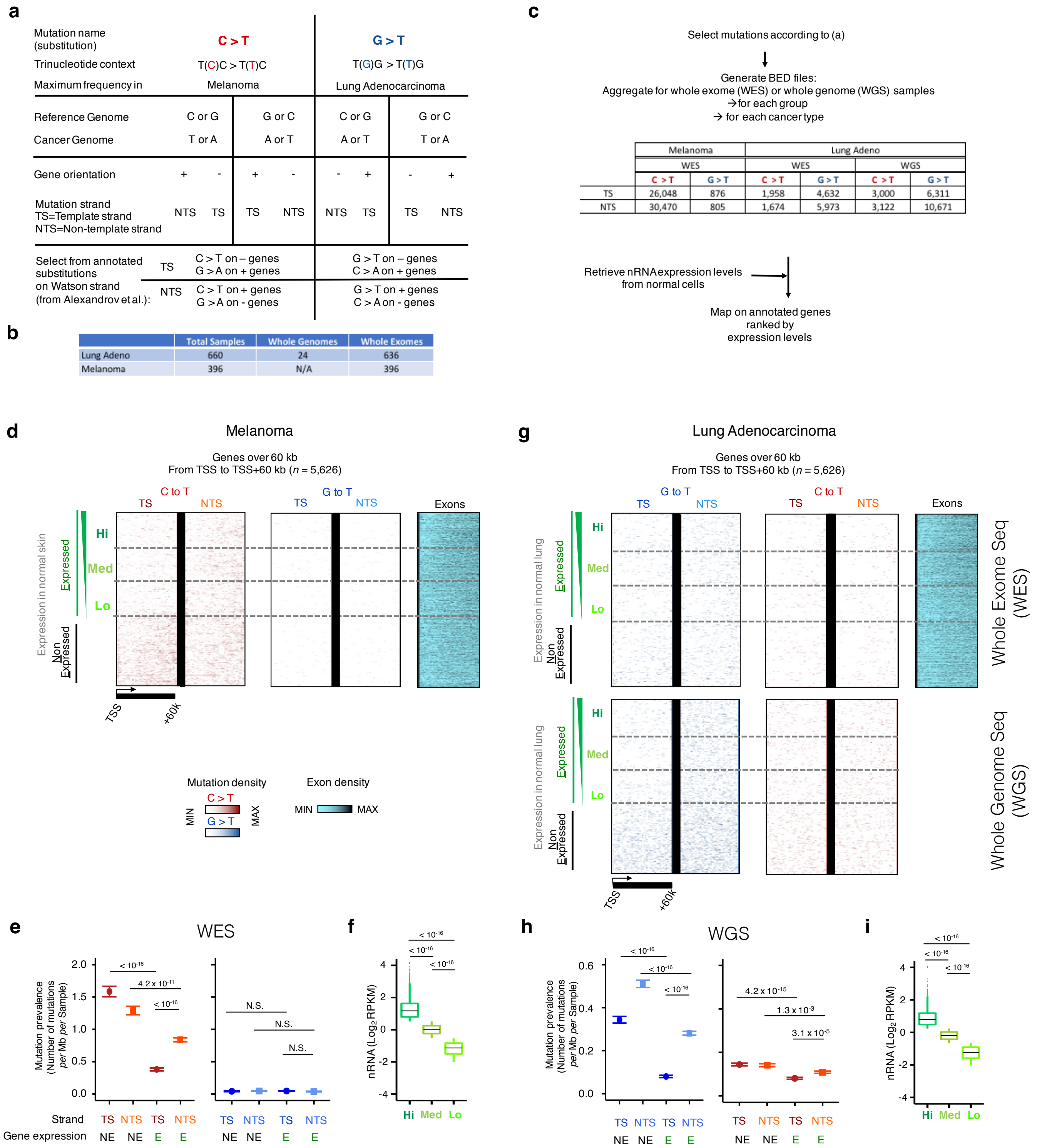

Supplementary Fig. 12 Mapping of NER-failure/the most frequent UV- and smoking- associated mutations highlights the role of stress-triggered RNAPII wave-release in shaping cancer genomes. **a** Table summarising the methodology used to extract most frequent UV- and smoking- associated mutations (in the indicated trinucleotide context) from published cancer genomes maps[2] of Melanoma and Lung Adenocarcinoma tumors. See also Methods. **b** Table summarising the number and type of samples analysed. **c** Scheme describing the procedure used to map the mutations obtained in (**a**) on gene bodies (from TSS to TSS +60 kb). See Methods for details. **d** Heatmaps of the density of mutations detected in a melanoma genome by WES as defined in (**a**-**c**) for template (TS) and non-template (NTS) strands separately. T(C)C > T(T)C (labeled as (C > T)) are substitutions highly associated with UV damage, while T(G)G > T(T)G (labeled as (G > T)) are highly associated with smoking damage. Genes are ranked by increasing expression levels (RPKM) as determined by nascent RNA-seq in normal fibroblasts and divided in expression quantiles as indicated. Exons density maps are also shown on the right. See also Methods. **e** Average (± SEM) mutation prevalence (number of mutations counted *per* Mb and *per* sample) for C > T and G > T in expressed and non-expressed genes in TS and NTS strands. Note: In WES analysis, given the non-linearity of exome data, we corrected mutation densities by the relative exon density measured in the considered regions. Wilcoxon rank-sum test was used to compare mutation prevalence scores. *P* values are indicated, N.S.: Non-Significant (N.S.) if $P < 0.05$. **f** Comparison of nRNA levels in expressed genes for the three expression quantiles defined above (two-sided t-test using the Benjaminin-Hoechberg adjustment was used to compare average RPKM values, *P* values are indicated). **g**-**i** Same as in (**d**-**f**) for lung adenocarcinoma genomes separately for WES and WGS samples. Note: WES- and WGS-extracted profiles were highly comparable. We chose to analyse further only WGS data because of a deeper and more continuous coverage (introns and exons), which did not require to further correct for relative exon density.

Supplementary Methods

Flow cytometry

Cell cycle analysis was achieved via Propidium Iodide (PI) staining. Cells were plated in 10 cm plates 48 h prior to serum-deprivation and were released or not from serum-deprivation by the addition of FBS to a final concentration of 10 %. Cells were collected and fixed in a solution of PBS, 0.1% Glucose and 70% ethanol and were left for 1 day at -20 ^{o}C. After fixation, the cells were centrifuged, washed once with PBS and stained with 50μg/ml PI (SIGMA). Next, 100μg/ml RNase A (Thermo Fisher Scientific) was added and the cells were immediately transferred into the appropriate FACS tubes and were incubated in a platform shaker in the dark for 40 minutes. PBS was added to terminate the reaction. Signals were acquired and analysed with FACSDiva software (Version 6.0; BD Biosciences) using a FACS Canto II flow cytometer (BD Biosciences). Percentages of cells in each cell cycle phase were determined for cells starved (+0.5% FBS) for 72 h and for cells released in complete medium (+10% FBS) for 3 to 5 h.

ChIP-seq

At least two independent cultures of cells per condition were cross-linked with 1% (v/v) formaldehyde for 10 min at room temperature in culture media, and the reaction was stopped by the addition of glycine (125 mM final). Cells were washed three times with PBS and resuspended in 1,5 ml of Cell Lysis Buffer (5 mM HEPES pH 8.0, 85 mM KCl, 0.5% NP-40, 1 mM PMSF (Sigma, 11359061001), protease inhibitors (from Sigma-Aldrich and used as recommended by the manufacturer; E-64 (E3132), Bestatin (10874515001), Aprotinin (10981532001), Trypsin-Chymotrypsin (T9777), Leupeptin (11034626001), Pepstatin (11359053001) and 10 mM Sodium pyrophosphate tetrabasic decahydrate (NaPy; S6422)) at ~$1.3x10^7$ cells/ml and incubated for 10 min at 4°C. Cell nuclei were collected by centrifugation at 2500 rpm for 8 min at 4°C and resuspended in Nuclei Lysis Buffer (50 mM Tris-Cl pH 8.1, 10 mM EDTA (pH 8.0), 1% SDS, 1 mM PMSF, protease inhibitors) at 0.4x107 nuclei/ml. Samples were sonicated using the Bioruptor water bath sonicator (Diagenode) using the high setting with 30 sec on and 30 sec off, for 30 minutes to generate 100-400 bp DNA fragments. Approximately 100-150 μg of chromatin (DNA concentration measured by NanoDrop) was used for each ChIP. Samples were diluted at least five times in IP dilution Buffer (0.01% SDS, 1.1% Triton X100, 1.2 mM EDTA (pH 8.0), 16.7 mM Tris-Cl, pH 8.1, 167 mM NaCl, 1 mM PMSF, protease inhibitors). Inputs were saved at 4°C until the next day to be reverse-crosslinked with ChIP samples. For the ChIP step, 2-4 μg of antibody were incubated with sheared chromatin suspension for 16 h at 4°C in the presence of 6-12 μL of blocked protein A or G Dynabeads (Thermo Scientific) and competitors (Bovine Serum Albumin (BSA) (30 μg/ml) and t-RNA (50 μg/ml)). The beads were washed sequentially with the following buffers: Low Salt Wash Buffer (0.1% SDS, 1% Triton X 100, 2 mM EDTA, 20 mM Tris-HCl pH 8, 150 mM NaCl, 1 mM PMSF, protease inhibitors), High Salt Wash Buffer (0.1% SDS, 1% Triton X 100, 2 mM EDTA, 20 mM Tris-HCl pH 8, 500mM NaCl, 1 mM PMSF, protease inhibitors), LiCl Wash Buffer (100 mM Tris-HCl, pH 7.5, 250 mM LiCl, 1% NP-40,

1% Sodium Deoxycholate, 1 mM EDTA, 1 mM PMSF, protease inhibitors), and two times with TE (10 mM Tris-HCl ph 8.0, 1 mM EDTA pH 8.0). Chromatin immunocomplexes were eluted twice by incubation with 1% SDS and 100 mM NaHCO3 at 65°C for 15 min with vortexing. Cross-linking of ChIP and Input material was reversed by adjusting to 200 mM NaCl and incubating samples for 12-16 h at 65°C. After Proteinase K treatment (0.2 μg/μl in 0.5 X TE) for 1h at 55°C, DNA was purified using the Ampure XP Beads (Agencourt) according to manufacturer's protocol without size selection (using a bead-to-sample ratio of 1.8).

Chromatin fractionation, immunoprecipitation and western blot

Pellets of ~2x10^7 cross-linked cells (see above in ChIP section) were resuspended in Chro-IP lysis Buffer (50 mM Hepes-KOH pH 8.0, 1 mM EDTA, 0.5 mM EGTA, 140 mM NaCl, 10% glycerol, 0.5% IGEPAL, 0.25% Triton X-100, 1 mM PMSF, and protease inhibitors as indicated above). After 10 min rotation at 4°C, the cell suspension was centrifuged (10 min, 2,800 rpm at 4°C) and the supernatant was kept as soluble fraction. In turn, cell pellet was washed with Wash Buffer (10 mM Tris-HCl, pH 8.0, 1 mM EDTA, 0.5 mM EGTA, 200 mM NaCl, 1 mM PMSF, 10 mM NaPy and protease inhibitors). The cell suspension was rotated for 10 min at 4°C and centrifuged as mentioned above. The cell pellet was resuspended in RIPA Buffer (10 mM Tris-HCl, pH 8.0, 1 mM EDTA, 0.5 mM EGTA, 140 mM NaCl, 1% Triton X-100, 0.1% Na-Deoxycholate, 0.1% SDS, 1 mM PMSF, 10 mM NaPy and protease inhibitors). Samples were sonicated (as described above) and centrifuged for 10 min at 10,000 rpm at 4°C. The supernatant was kept as chromatin fraction. Equal amounts of chromatin were either subjected directly to western blot analysis (Input) or immunoprecipitated after overnight incubation at 4°C with ~2 μg of an antibody against pan-RNAPII (Rpb1 N-terminus) (EPR1509Y; ab76123, lot# YI03010C). Next day, the immunoprecipitated chromatin was incubated with Protein A beads (Dynabeads) for 3 h at 4°C. The beads with the bound chromatin immunocomplexes were then washed as described above (ChIP Section). Western Blot analysis of equal amounts of chromatin fraction material (Input) or equal volume of beads suspension (ChIP) and 2x Laemmli sample buffer were mixed before incubation for 45min at 95°C and subjected to SDS-PAGE Western blot as previously described[3] using Odyssey-compatible IRDye680- and IRDye800- conjugated secondary antibodies and according to manufacturer's instruction (Odyssey CLx Infrared Imaging System, LI-COR Biosciences). Experiments were conducted in two biological replicates. We quantified bands intensity in all isoforms and normalised the signal detected in the recovery time points to NO UV (steady–state). Average percentage signal is given for each isoform (± SEM). Antibodies used for western blots are RNAPII CTD (phospho-S2) (dilution 1: 1,000; ab5095, lot GR231750-2) from abcam; RNAPII CTD (phospho-S5) (3E8; dilution 1: 1,000; 04-1572-I, lot: 2395817) and RNAPII CTD (hypophosphorylated) (8WG16; dilution 1: 500; 05-952, lot: 2262610) from Millipore and loading controls were estimated by levels of histone H3 (dilution 1: 10,000). Results shown are representative of three independent biological replicates.

Damaged-DNA immunoblot analysis (dot- or slot-blot)

ChIPed and Input DNA purified and quantified by Qubit assay (as detailed in the 'ChIP' section) were denatured by boiling for 5 min and placing on ice in a final concentration of 6 x SSC buffer. Samples were then spotted onto a washed (with TE) LI-COR Odyssey Nitrocellulose membrane (LI-COR Biosciences) using the MINIFOLD I slot-blot system as previously described with minor modifications[3]. Wells were then washed in 6 x SSC + 0.7 x TE buffer three times. Membrane was incubated at 80°C for 2 h and blocked for 1 h with a 1:1 solution of PBS and Odyssey Blocking Buffer (OBB, LI-COR Biosciences). The analysis was performed as described with minor modifications: the membrane was incubated overnight at 4°C with an antibody against CPDs (CosmoBio USA, CAC-NM-DND-001, Lot: TM C-05), and washed with PBS:OBB (1:1) buffer. The signal intensities were recorded as above for Western blots and quantified using the Image Studio software (LI-COR Biosciences). Blots shown are representative of at least two independent biological replicates.

mRNA-seq

Total RNA from two independent cultures of serum-starved synchronised and released in 10% FCS cells was extracted using Trizol reagent (Life Technologies) and genomic DNA was removed with Turbo DNAse (Ambion). The polyA fraction (mature mRNA) was isolated by running RNA samples two times through the mRNA purification kit (NEBNext Oligo d(T)25) to ensure higher purity. The resulting RNA fractions were used for mRNA-seq library preparation. Briefly, fragmentation at 94°C for 4 min was carried on in 2 x First strand synthesis buffer (FSS) from the SuperScript II kit (Life Technologies). RNAs were then incubated with random hexamer (Life Technologies) and SuperScript II enzyme in 1 x FSS containing DTT (10 mM) in accord with manufacturer's guidelines. Second strand cDNA synthesis was carried out directly after dilution of the First strand synthesis reaction in the presence of 24 U DNA polI, 20 U RNase H and dNTPs (Life Technologies) in 1 x Second Strand Synthesis Buffer (NEBNext) for 2 h at 16°C. Double stranded (ds) cDNA was size-selected and purified (Agencourt Ampure XP beads, Beckman) and each biological replicate was used for library preparation.

Nascent RNA fluorescence microscopy (EU-Fluo)

For fluorescence microscopy of nascent RNA (EU-Fluo), cells were incubated with EU (0.1 mM) in duplicates for 120 min at different times before and after UV irradiation as indicated in the Figure legend, and as previously described[4]. Briefly, cells were washed with PBS after EU incubation, fixed and permeabilised. Click-IT was performed for 1 h at RT in the presence of 25 µM Alexa Fluor® 594 azide, 10 mM sodium ascorbate, 4 mM Copper (II) sulfate and RNaseOUT (Thermo Scientific). Results shown are representative of three independent biological replicates.

DRB-inhibition (preDRB-ChIP-seq)

For preDRB-ChIP-seq, cells were pre-incubated with DRB (100 µM) for 12 h, then chromatin was cross-linked and isolated (see 'ChIP' Section) after 1 h of recovery from UV (20 J/m^2) or mock treatment (DMSO) in complete medium, and RNAPII-ser2P was immunoprecipitated.

CPDIP-seq

Mapping of CPD lesions was achieved through modification of MeDIP-seq protocol (https://www.epigenesys.eu/images/stories/protocols/pdf/20111026125309_p33.pdf). Briefly, cells were crosslinked immediately after UV irradiation (8 J/m^2) and sheared Input DNA was purified as described in the 'ChIP' section. Purified dsDNA was denatured and the resulting single stranded (ss)DNA was immunoprecipitated as follows. Protein G dynabeads were washed and blocked in PBS-BSA 0.1% ('ChIP' section) before being incubated with anti-CPD antibody (Anti Cyclobutane Pyrimidine Dimers, clone TDM-2, cat: NMDND001, lot TMC-05) and ssDNA for 2 h at 4°C in 1 x CPDIP buffer (10 mM Na-Phosphate buffer pH 7.0 (1 M stock was prepared fresh as follows: 39 ml of 2 M monobasic sodium phosphate (NaH_2PO_4) (276 g/L) was added to 61 ml of 2 M dibasic sodium phosphate (Na_2HPO_4)(284 g/L)), 140 mM NaCl, 0.05 % Triton X-100). Samples were then washed in CPDIP buffer three times and DNA was eluted and precipitated. Dot-blot was performed to measure the enrichment of pulled-down DNA in UV-photolesions (CPDs) as compared to the levels of CPD detected in similar or indicated amounts of Input DNA (Qubit, see Figure legends). CPDIP bound and Input DNA were checked by qPCR analyses (data not shown) performed in duplicate reactions (see ChIP Section). Remaining amounts of CPDIP-material were then used for library preparation and sequencing after ssDNA was transformed to dsDNA as described in mRNA-seq section.

Reads alignment and normalisation

ChIP-Seq, CPDIP-seq, and Input-seq reads were quality trimmed and Illumina adaptors were removed with the cutadapt tool[5], keeping resulting reads of at least 20 bp (cutadapt -a -q and -m parameters). Reads were then aligned uniquely to the GRCh37/hg19 reference genome using bwa (version 0.7.12)[6] allowing up to two mismatches (bwa aln default parameters). To gain better specificity on the tag enriched regions and increase sensitivity, non-redundant reads were complemented with a fraction of redundant reads[7] for normalising each sample of the set analysed during the experiment. For simplicity, only the steady-state Input is shown and was used as a representative sample among all sequenced Inputs (high correlation scores, data no shown).

For nRNA-seq, reads were also quality trimmed and adaptor clipped as above. To remove rRNA reads, remaining reads were first aligned to the human ribosomal DNA complete repeating unit (U13369.1) allowing up to 1 mismatch using Bowtie[8] (version 1.1.0) (bowtie -n 1 -k 1 -m 1 and --un parameters), and the unmapped reads were aligned uniquely to the GRCh37/hg19 reference genome allowing up to 2 mismatches (bowtie -n 2 -k 1 and -m 1 parameters). For nRNA-seq both redundant and non-redundant intronic reads were considered in order to increase the dynamic range of our analysis and to limit the background of unlabeled mature RNA[9,10]. For preDRB-nRNA-seq (with DRB treatment) experiments, only non-redundant intronic reads were considered (sort -k1,1 -k2g,2 -u unix command) to limit the effect of variability in transcript coverage possibly generating artifacts during PCR amplification of the libraries as previously suggested[11,12]. For mRNA-seq, reads were mapped with Tophat 2.0.12[13] (tophat2 --library-type fr-unstranded --keep-

fasta-order --bowtie1 -GTF parameters). RPKM values were calculated with the 'rpkm ()' function of the Bioconductor R package edgeR (3.14.0)[14] using exon length (as *per* our exon annotation) for mRNA-seq and gene length for nRNA-seq (as *per* our transcript annotation) (see Methods). Sequenced, mapped and normalised read stats are depicted in Supplementary Data 1. BigWig files were generated using samtools (1.2)[15] (`samtools view -bS`), BEDTools (2.22.1)[16] (`bedtools bamtobed`, `bedtools genomecov -bg -i -g`) and UCSC tools[17] (`bedGraphToBigWig`) to produce tracks compatible with UCSC genome browser and export snapshots of the normalised data presented throughout the figures.

Supplementary References

1. Hu, J., Adar, S., Selby, C. P., Lieb, J. D. & Sancar, A. Genome-wide analysis of human global and transcription-coupled excision repair of UV damage at single-nucleotide resolution. *Genes Dev.* **29,** 948–960 (2015).
2. Alexandrov, L. B. *et al.* Signatures of mutational processes in human cancer. *Nature* **500,** 415–421 (2013).
3. Tresini, M. *et al.* The core spliceosome as target and effector of non-canonical ATM signalling. *Nature* **523,** 53–58 (2015).
4. Ogi, T. *et al.* Three DNA Polymerases, Recruited by Different Mechanisms, Carry Out NER Repair Synthesis in Human Cells. *Mol. Cell* **37,** 714–727 (2010).
5. Martin, M. Cutadapt removes adapter sequences from high-throughput sequencing reads. *EMBnetjournal Vol 17 No 1 Gener. Seq. Data Anal.* (2011).
6. Li, H. & Durbin, R. Fast and accurate short read alignment with Burrows-Wheeler transform. *Bioinformatics* **25,** 1754–1760 (2009).
7. Chen, Y. *et al.* Systematic evaluation of factors influencing ChIP-seq fidelity. *Nat. Methods* **9,** 609–614 (2012).
8. Langmead, B., Trapnell, C., Pop, M. & Salzberg, S. L. Ultrafast and memory-efficient alignment of short DNA sequences to the human genome. *Genome Biol* **10,** (2009).
9. Core, L. J. & Lis, J. T. Transcription Regulation Through Promoter-Proximal Pausing of RNA Polymerase II. *Science* **319,** 1791–1792 (2008).
10. Veloso, A. *et al.* Rate of elongation by RNA polymerase II is associated with specific gene features and epigenetic modifications. *Genome Res.* (2014). doi:10.1101/gr.171405.113
11. Wang, L. *et al.* Molecular basis for 5-carboxycytosine recognition by RNA polymerase II elongation complex. *Nature* **523,** 621–625 (2015).
12. Laitem, C. *et al.* CDK9 inhibitors define elongation checkpoints at both ends of RNA polymerase II–transcribed genes. *Nat. Struct. Mol. Biol.* (2015). doi:10.1038/nsmb.3000
13. Kim, D. *et al.* TopHat2: accurate alignment of transcriptomes in the presence of insertions, deletions and gene fusions. *Genome Biol.* **14,** 1–13 (2013).
14. Robinson, M. D., McCarthy, D. J. & Smyth, G. K. edgeR: a Bioconductor package for differential expression analysis of digital gene expression data. *Bioinformatics* **26,** 139–140 (2010).
15. Li, H. *et al.* The Sequence Alignment/Map format and SAMtools. *Bioinformatics* **25,** 2078–2079 (2009).
16. Quinlan, A. R. & Hall, I. M. BEDTools: a flexible suite of utilities for comparing genomic features. *Bioinformatics* **26,** 841–842 (2010).
17. Kent, W. J., Zweig, A. S., Barber, G., Hinrichs, A. S. & Karolchik, D. BigWig and BigBed: enabling browsing of large distributed datasets. *Bioinformatics* **26,** 2204–2207 (2010).
