# Supplementary material for: Global unleashing of transcription elongation waves in response to genotoxic stress restricts somatic mutation rate
Source: Nat Commun. 2017 Dec 12;8:2076. doi: 10.1038/s41467-017-02145-4 (PMC5727188; doi:10.1038/s41467-017-02145-4)
Supplement: Supplementary file 3 — Description of Additional Supplementary Files [file 41467_2017_2145_MOESM3_ESM.docx]

**Description of Additional Supplementary Files**

File Name: Supplementary Data 1

Description: Table describing the summary of the details for all sequenced samples in the study.

File Name: Supplementary Data 2

Description: Table describing ChIP-seq peaks information.

File Name: Supplementary Data 3

Description: Table describing gene annotation and expression status details.
